# Twins Discordant for Parkinson’s Disease: A 10-Year Longitudinal Follow-Up of Mortality, Clinical and Lifestyle Features, Nutritional Status, and Dietary Habits

**DOI:** 10.3390/brainsci16101102

**Published:** 2026-10-13

**Authors:** Michela Barichella, Antonio Natale, Elena Contaldi, Luca Magistrelli, Emanuele Cereda, Carlotta Bolliri, Maria Carmela Macchione, Federica Invernizzi, Daniela Calandrella, Ioannis Ugo Isaias, Gianni Pezzoli

**Affiliations:** 1 Clinical Nutrition Unit, ASST Gaetano Pini-CTO, 20126 Milan, Italy; 2 Fondazione Pezzoli per la Malattia di Parkinson—ETS, 20126 Milan, Italy; 3 Parkinson Institute of Milan, ASST Gaetano Pini-CTO, 20126 Milan, Italyluca.magistrelli@asst-pini-cto.it (L.M.); 4 Clinical Nutrition and Dietetics Unit, Fondazione IRCCS Policlinico San Matteo, 27100 Pavia, Italy; e.cereda@smatteo.pv.it; 5 Center for Liver Disease, IRCCS Ospedale San Raffaele, 20132 Milan, Italy

**Keywords:** Parkinson’s disease, discordant twins, longitudinal study, mortality, nutritional status, dietary habits

## Abstract

**Highlights:**

- Ten-year follow-up of 54 PD-discordant twin pairs (26 monozygotic).
- Mortality was ~7-fold higher in affected twins (HR 6.83); all MZ deaths occurred in the affected twin.
- Excess mortality persisted despite no detected within-pair differences in BMI or comorbidities.
- Constipation was more frequent in affected twins at both time points, and within MZ pairs at follow-up.
- Fruit intake increased over time in affected twins, possibly as an adaptation to constipation.

**Abstract:**

**Background/Objectives**: Twins discordant for Parkinson’s disease (PD) share genetic background and early-life exposures, offering a model for non-genetic determinants of disease. We aimed to evaluate longitudinally the mortality, clinical and lifestyle features, nutritional status and dietary habits of a cohort of PD-discordant twins, with specific attention to the monozygotic (MZ) subgroup. **Methods**: One hundred and eight subjects from 54 PD-discordant twin pairs (26 MZ, 28 dizygotic [DZ]), with genetically confirmed zygosity, were assessed at baseline (T0) and after 10 years by telephone interview (T1; 33 pairs), using Kaplan–Meier/Cox survival analyses, a clinical and lifestyle history questionnaire, body mass index (BMI), and a food frequency questionnaire (FFQ). **Results**: Ten-year mortality was markedly higher in PD (31.5% vs. 5.6%; HR 6.83), with all MZ deaths occurring in the affected twin. Constipation was more frequent in PD at both time points (T0 *p* = 0.004; T1 *p* = 0.011). Comorbidities, smoking, weight and BMI did not differ within pairs. Preserved-fish intake was stably lower in PD (T0 *p* = 0.021; T1 *p* = 0.004); fruit intake was lower in PD at baseline (MZ, *p* = 0.001) but higher at follow-up (*p* = 0.029). **Conclusions**: The mortality difference persisted despite no detected within-pair differences in BMI and comorbidities, consistent with a major contribution of PD itself. Preserved-fish intake remained stably lower in affected twins, whereas increased fruit intake at follow-up may reflect adaptation to constipation; these exploratory dietary findings warrant confirmation.

## 1. Introduction

Parkinson’s disease (PD) is the second most common neurodegenerative disorder and the fastest-growing in prevalence and disability, a trend driven by population ageing. Its aetiology is multifactorial, reflecting a complex interplay between genetic susceptibility and environmental exposures, and disentangling these components is one of the central challenges of PD epidemiology. The twin model is uniquely suited to this task: monozygotic (MZ) twins share 100% of their genetic material and dizygotic (DZ) twins about 50%, while both share the intrauterine environment, early nutrition and much of their socioeconomic context. Within a pair discordant for PD, therefore, the difference cannot be attributed to inherited DNA alone and instead points to differential environmental or behavioural exposures accumulated over the life course, or to a different latency of disease expression [1,2,3,4,5,6]. Long-term follow-up of such pairs, however, remains scarce [7,8,9,10].

Several modifiable factors have been repeatedly implicated in PD, including dietary habits, cigarette smoking and occupational exposure to pesticides and organic solvents [11,12,13,14,15]. Nutritional status is also clinically relevant: malnutrition and involuntary weight loss are frequent in PD and track with disease severity and quality of life [16,17]. Among non-motor features, gastrointestinal dysfunction—particularly constipation—is one of the earliest manifestations and is hypothesised to reflect early involvement of the enteric nervous system within the gut–brain axis, in line with the Braak staging hypothesis [18,19,20].

The twin model has been investigated in PD for several decades: studies have analysed the National Research Council World War II Veteran Twins Registry, identifying 193 twins with PD [6]; a nationwide Finnish cohort, identifying 42 PD cases [5]; and a 20-year follow-up study of concordance and heritability involving more than 200 twin pairs [9]. However, these studies primarily used twin pairs—particularly monozygotic twins—as a unique model to investigate the contribution of environmental and non-genetic factors to PD. Because monozygotic twins share virtually the same genetic background, differences in disease occurrence can be more confidently attributed to environmental, lifestyle or stochastic influences rather than to genetic variation. Among lifestyle-related exposures, cigarette smoking has emerged as the most consistently replicated association. In one of the earliest case–control studies of 44 PD-discordant twin pairs (31 monozygotic and 13 dizygotic), the affected twin had generally smoked less than the unaffected co-twin [1], an inverse association between smoking and PD that was subsequently confirmed in Swedish twins [7].

More recently, research on PD-discordant monozygotic twins has expanded beyond estimating the relative contribution of genetic and environmental factors to investigate molecular mechanisms that may explain disease discordance despite a shared genetic background. Cross-sectional studies have explored several biological markers, including gut microbiome composition, epigenetic modifications, and telomere length. Within this framework, our group investigated gut microbiota alterations [21] and leukocyte telomere length [22] in monozygotic twins discordant for PD, providing further insights into non-genetic mechanisms potentially involved in disease pathogenesis. However, all these investigations were cross-sectional and assessed molecular or clinical features at a single time point, leaving the longitudinal evolution of these markers and phenotypes largely unexplored.

What remains lacking is a longitudinal within-pair follow-up. To the best of our knowledge, no previous twin study has prospectively investigated longitudinal changes in mortality, clinical and lifestyle characteristics, nutritional status and dietary habits among twins discordant for PD. The primary aim of the present study was therefore to compare 10-year mortality between affected twins and their unaffected co-twins in a cohort of PD-discordant twin pairs, with specific attention to the MZ subgroup. Secondary, exploratory aims were to describe within-pair differences in clinical and lifestyle features, nutritional status and dietary habits at baseline and, in the pairs reassessed after 10 years, their evolution over time.

## 2. Methods

### 2.1. Study Design

This study combined a cross-sectional baseline (T0) with a 10-year longitudinal follow-up (T1) at the Parkinson Centre, ASST Gaetano Pini-CTO, Milan. Through a national campaign promoted by the Fondazione Pezzoli per la Malattia di Parkinson (2015–2017), 73 twin pairs with at least one member affected by parkinsonism were recruited. For the present analysis, 54 pairs discordant for idiopathic PD (26 MZ, 28 DZ; 108 subjects) were selected, excluding atypical parkinsonian syndromes and essential tremor. Within each pair, one twin met established clinical criteria for PD [23], while the co-twin was free of neurodegenerative disease. Zygosity was determined by genetic testing based on microsatellite analysis. At baseline (T0), clinical and lifestyle data (disease duration, levodopa dose, non-neurological comorbidities, gastrointestinal symptoms, and smoking habits) were collected through structured questionnaires administered to participants who attended the Parkinson Centre of ASST Gaetano Pini-CTO, Milan, in response to the national campaign described above, and who therefore underwent a specialist assessment. Body weight and height were measured directly using a calibrated scale and stadiometer, respectively. Body mass index (BMI) was calculated by dividing body weight (in kilograms) by the square of height (in metres). Dietary habits were assessed using a food frequency questionnaire (FFQ), developed by adapting a questionnaire previously used by our research group [24]. The FFQ was non-quantitative and recorded the habitual frequency of consumption of the main food groups, expressed as food consumption frequency scores. The FFQ included separate items for fresh fish and preserved fish; the latter comprised canned fish preserved in oil (tuna, mackerel and sardines) and smoked salmon. At the 10-year follow-up (T1), all 108 subjects were contacted by telephone between January and March 2026. Vital status was verified first; then, among participants who agreed to continue in the study, within-pair discordance was reassessed. A structured telephone interview based on the same questionnaire was conducted to collect updated information on the clinical and lifestyle data assessed at baseline (disease duration, levodopa dose, non-neurological comorbidities, gastrointestinal symptoms, and smoking habits), as well as on additional lifestyle-related factors, such as lifetime occupational and residential exposure to pesticides, herbicides, organic solvents, and heavy metals, which were therefore assessed retrospectively at T1 only. Because no direct anthropometric measurements were possible at T1, body weight was self-reported by the participants, and BMI was calculated using self-reported weight and the height measured at baseline. Current dietary habits were reassessed using the FFQ administered at baseline, adapted for a brief telephone interview.

### 2.2. Study Endpoints

Mortality was the primary longitudinal endpoint: deaths occurring during the follow-up period were ascertained through family contacts and, where available, by reviewing data from the electronic digital archive (AED) of the Parkinson Centre. Whenever possible, the year and cause of death were recorded. We also assessed whether previously unaffected co-twins had developed PD during the follow-up period. Because PD is a multisystem, slowly progressive condition whose broader impact is not captured by mortality alone, the clinical, nutritional and dietary variables described in Section 2.1 were analysed as secondary, exploratory endpoints. These analyses were not intended to identify independent predictors of mortality, but rather to characterise—within a genetically and environmentally matched design—whether factors previously linked to PD in the literature also diverge within discordant pairs over a decade, and whether any such divergence could plausibly contribute to, or instead be dissociated from, the observed mortality difference.

### 2.3. Study Population

**Figure 1:**
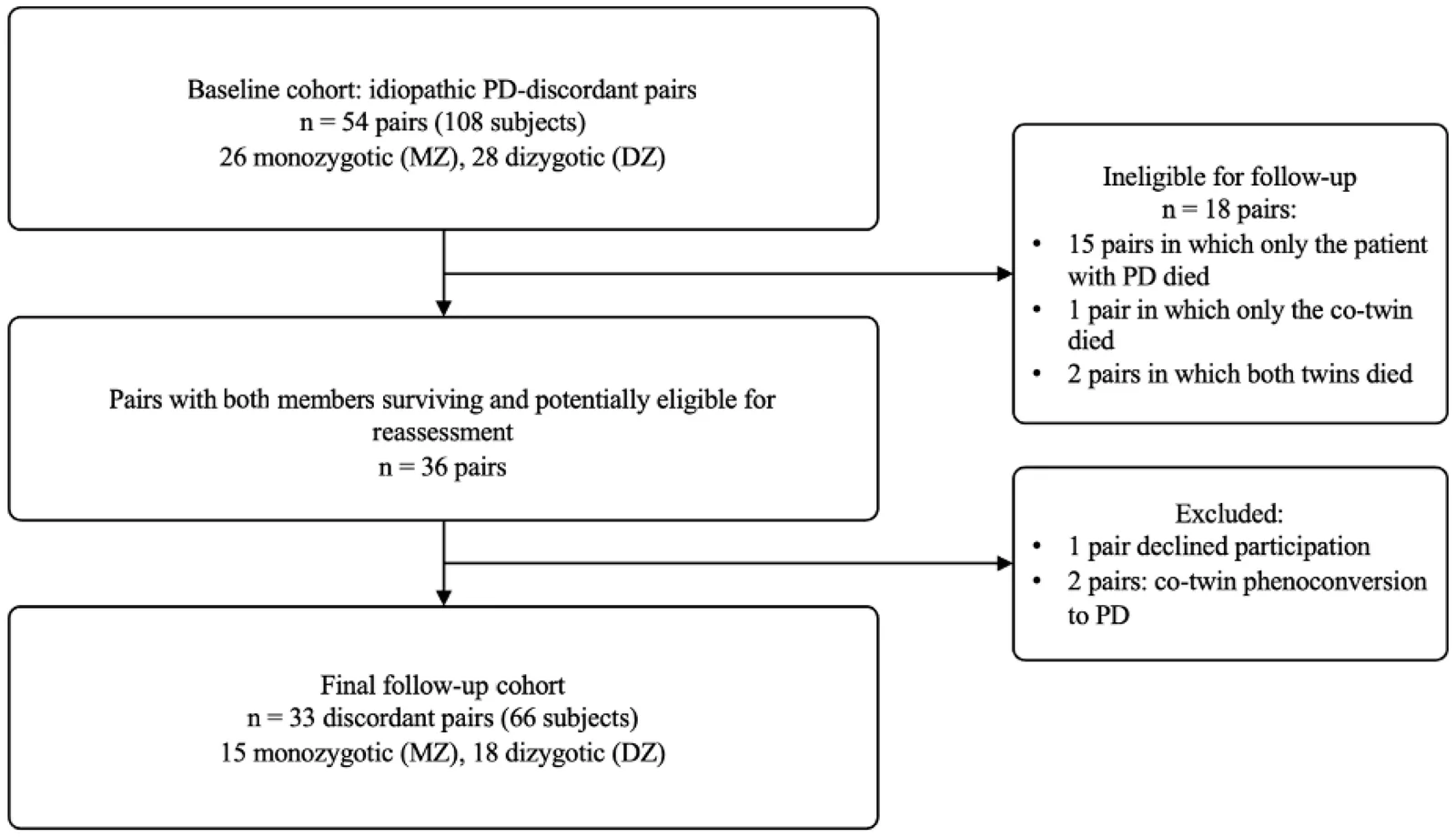
Flow diagram of participant selection. PD, Parkinson’s disease; MZ, monozygotic; DZ, dizygotic.

Baseline analyses included all 54 twin pairs discordant for idiopathic PD (108 participants; age 66.17 ± 10.82 years; 55.5% men, 44.5% women; 26 MZ and 28 DZ pairs), with additional analyses performed in the MZ subgroup to maximize genetic control. The 10-year follow-up was restricted to the idiopathic-PD cohort (Figure 1). Of the 54 pairs enrolled at baseline, 18 became ineligible for reassessment because at least one twin had died, leaving 36 pairs potentially eligible for follow-up. Of these, one pair declined to participate, and two MZ pairs were excluded because the initially unaffected co-twin had developed PD during follow-up (phenoconversion), thereby losing within-pair discordance (Section 3.5). The follow-up cohort therefore comprised 33 discordant pairs (15 MZ and 18 DZ; age at baseline 60.55 ± 9.42 years and at follow-up 70.45 ± 9.83 years; 50% men), i.e., all pairs that remained alive, discordant and available for reassessment. In one family enrolled as a triplet (one affected and two unaffected siblings), a single unaffected sibling was retained as co-twin. To evaluate attrition bias, the baseline characteristics of the 33 retained pairs were compared with those of the 21 pairs not retained (Supplementary Table S1).

### 2.4. Statistical Analysis

Continuous variables are summarised as mean ± standard deviation (SD) and categorical variables as n (%). Mortality, the primary endpoint, was analysed in all 54 pairs by the Kaplan–Meier method with the log-rank test, from the baseline visit to the year of death or, for survivors, to the follow-up interview (January–March 2026). Hazard ratios (HRs) for affected twins versus their unaffected co-twins were estimated by Cox regression with twin status as the covariate; to account for the matched design, a Cox model stratified by twin pair (HR estimated within pairs) was fitted as a sensitivity analysis. In the MZ subgroup, in which all deaths occurred in affected twins (complete separation), Firth’s penalized partial likelihood with profile penalized-likelihood confidence intervals (CIs) was used. Within-pair comparisons of continuous and FFQ variables were performed with the paired Student’s *t*-test, and all paired categorical comparisons, including deaths, with the McNemar test (asymptotic χ^2^ without continuity correction; the exact binomial McNemar was used where discordant pairs were few, e.g., sparse comorbidity cells and the MZ subgroup). In the 33 pairs reassessed at follow-up, within-pair differences were assessed at T0 and T1, within-group changes between T0 and T1 were assessed by paired tests, and differences in change between affected twins and co-twins by a paired *t*-test on within-pair differences. All analyses were repeated in the MZ subgroup. Normality of within-pair differences was assessed using the Shapiro–Wilk test, and all continuous/FFQ comparisons were repeated with the Wilcoxon signed-rank test as a sensitivity analysis (Supplementary Table S2). Secondary analyses were exploratory: the nominal *p*-values of the within-pair comparisons of the secondary endpoints were adjusted with the Benjamini–Hochberg false discovery rate (FDR) procedure within each domain (smoking, metabolic comorbidities, gastrointestinal symptoms, environmental exposure, nutritional status and dietary habits), separately for each time point and subgroup, and across all 78 comparisons (Supplementary Table S3). Baseline characteristics of retained and non-retained pairs were compared with Welch’s *t*-test and Fisher’s exact test (Supplementary Table S1). A two-sided *p* < 0.05 was considered statistically significant. Survival analyses were performed in R (R Foundation for Statistical Computing, Vienna, Austria) using the survival and coxphf packages.

### 2.5. Ethical Standards

This study was conducted in accordance with the Declaration of Helsinki; details of ethical approval and informed consent are provided in the Institutional Review Board Statement and Informed Consent Statement below.

## 3. Results

### 3.1. Mortality and Survival

**Figure 2:**
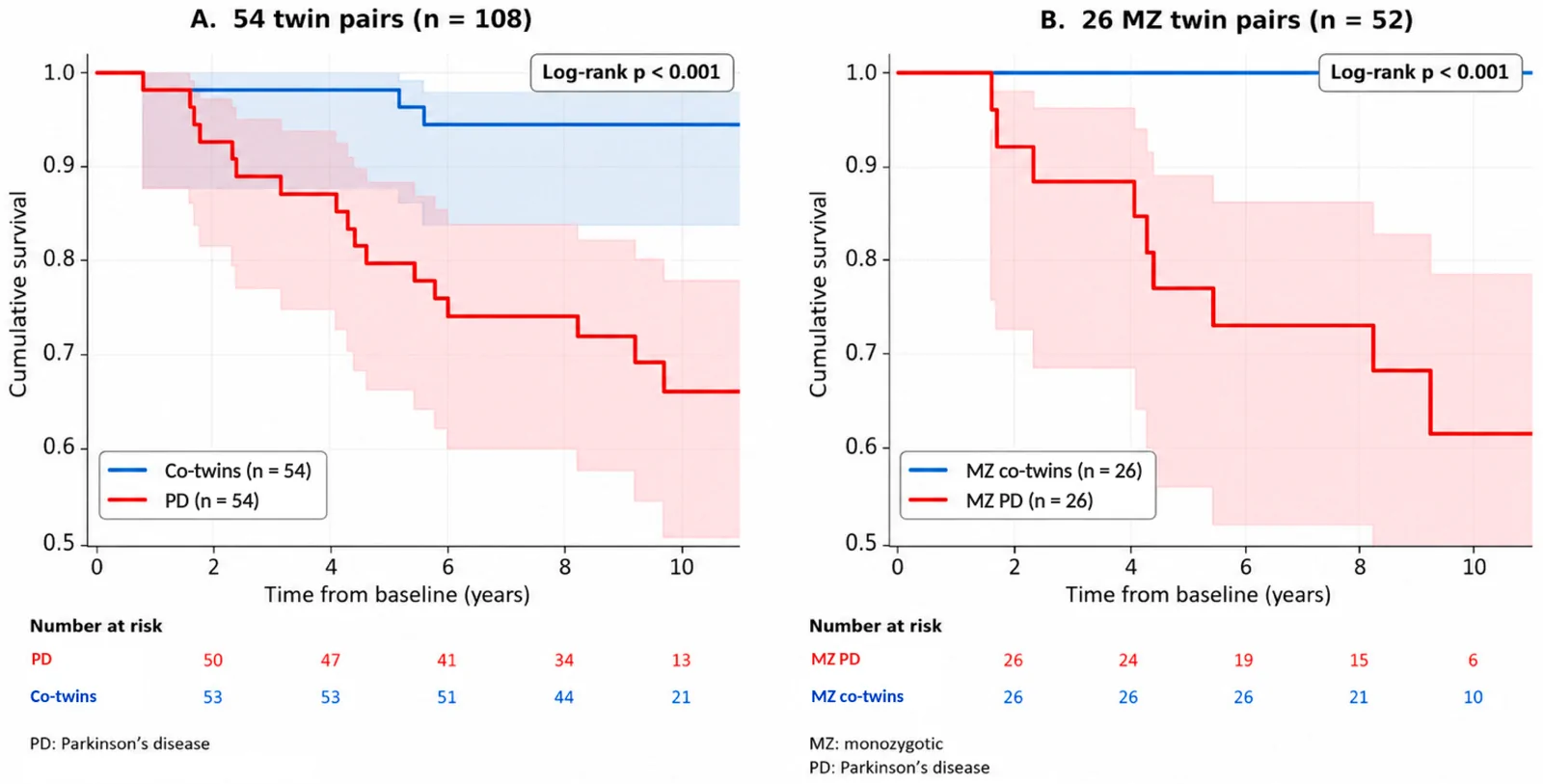
Kaplan–Meier survival curves in twins discordant for PD. (**A**) Whole sample (n = 108): 10-year survival 65.5% in the affected twins versus 94.5% in the co-twins (log-rank *p* < 0.001; hazard ratio 6.83, 95% CI 2.00–23.31). (**B**) Monozygotic subgroup (n = 52): 10-year survival 61.4% in the affected twins versus 100% in the MZ co-twins (log-rank *p* < 0.001). Numbers at risk are shown below each panel. PD, Parkinson’s disease; MZ, monozygotic; HR, hazard ratio; CI, confidence interval.

Mortality was the primary longitudinal endpoint and showed the largest difference between groups. Over the 10-year follow-up, 17 of the 54 twins with PD died compared with 3 of the 54 unaffected co-twins (McNemar *p* = 0.0005). The pattern was even more pronounced when stratified by zygosity: among the 26 monozygotic pairs, all 9 deaths occurred in the affected twin and none in the genetically identical co-twin, whereas among the 28 dizygotic pairs, 8 affected twins and 3 co-twins died. Mortality was higher in the affected twin in both sexes. Among men, 11 of the 32 affected twins died compared with 3 of the 27 co-twins (log-rank *p* = 0.039), while among women, 6 of the 22 affected twins died, compared with none of the 27 co-twins (log-rank *p* = 0.004), confirming that the within-pair survival disadvantage was present in both men and women. Among affected twins, the most frequent causes of death were complications of the neurodegenerative disease (n = 5), including one case of aspiration pneumonia secondary to dysphagia. Other causes of death included cardiovascular events (n = 3), COVID-19 (n = 1), cancer (n = 1), and other or unspecified causes (n = 6). Among the three deceased co-twins, one died from cancer, whereas the other two deaths were due to unspecified causes. At T1, both members of two pairs had died, including one pair already aged 91 years at baseline (deaths at 94 and 96 years); all deaths were included in all analyses. Kaplan–Meier analysis confirmed a significant divergence in cumulative survival between affected twins and their co-twins (Figure 2A; log-rank χ^2^ = 12.64, *p* < 0.001). Cox regression yielded an HR of 6.83 (95% CI 2.00–23.31; *p* = 0.002) for affected twins compared with their unaffected co-twins. The result was consistent in the sensitivity analysis accounting for the matched design: in the Cox model stratified by twin pair, the HR was 8.00 (95% CI 1.84–34.79; *p* = 0.006). Given the limited number of events, all CIs were wide, and the magnitude of the excess risk should be interpreted with caution. This divergence was even more pronounced in the monozygotic subgroup (Figure 2B), with highly significant differences in survival (log-rank *p* < 0.001). Because all nine MZ deaths occurred in the affected twin (zero events among MZ co-twins), the standard Cox partial likelihood is not estimable in this subgroup (complete separation); a Firth penalized-likelihood Cox model, which remains stable under this condition, yielded HR 22.95 (95% CI 2.90–2962.10; *p* = 0.0007; Table 1). The direction of this estimate is consistent with higher mortality in affected twins, but its magnitude is highly uncertain because of the small number of events and the complete separation. The estimated survival percentages at the different follow-up time points are reported in Table 1. BMI did not differ between twins who died and those who survived, either in the overall cohort (23.6 ± 4.7 vs. 24.8 ± 4.6; *p* = 0.28) or among affected twins (23.2 ± 4.9 vs. 25.1 ± 5.3; *p* = 0.19). Likewise, the point-biserial correlation between baseline BMI and mortality was weak and not statistically significant in the overall cohort (r = −0.11, *p* = 0.27), indicating that the excess mortality was not explained by baseline nutritional status.

### 3.2. Clinical and Lifestyle Features

Disease duration and levodopa dose both increased substantially from baseline to follow-up, consistent with the expected progression of PD (Table 2). The baseline values of the 33 pairs reassessed at follow-up are shown in the middle columns of Table 2, Table 3 and Table 4, allowing changes over time to be assessed within the same pairs; compared with the pairs not retained, these pairs were younger and, among affected twins, had shorter disease duration at baseline (Supplementary Table S1).

Active smoking was more frequent in unaffected co-twins than in affected twins, consistent with the inverse association between smoking and PD reported in the literature, although the difference was not statistically significant (*p* = 0.22). Among the pairs followed up, smoking status changed little over time (active smokers: from 2 to 2 affected twins and from 4 to 2 co-twins). The most consistent and clinically meaningful within-pair difference involved gastrointestinal symptoms. Constipation was significantly more frequent in affected twins than in their unaffected co-twins both at baseline (*p* = 0.004) and at follow-up (*p* = 0.011), when the difference was also significant within the genetically identical MZ pairs (*p* = 0.031); correspondingly, a regular bowel habit was less common in affected twins (*p* = 0.008 at T0), a difference that also became significant within the MZ subgroup at follow-up (26.7% vs. 73.3%; *p* = 0.016). In the pairs followed up, these differences reflected a worsening in affected twins over time: from T0 to T1, constipation increased from 27.3% to 48.5% (within-group change, *p* = 0.039) and a regular bowel habit decreased from 66.7% to 42.4% (*p* = 0.021), with no significant change in co-twins. These gastrointestinal differences remained significant after FDR correction within the gastrointestinal domain (q ≤ 0.047; Supplementary Table S3). Dysphagia, absent at baseline, emerged almost exclusively in affected twins at follow-up. Metabolic comorbidities did not differ within pairs at baseline, but hypertension tended to be more frequent in the unaffected co-twins at follow-up (48.5% in co-twins vs. 30.3% in affected twins; *p* = 0.109). In the pairs followed up, comorbidities did not differ within pairs at baseline either (affected twins vs. co-twins: hypertension 24.2% vs. 18.2%, *p* = 0.63; cardiovascular disease 21.2% vs. 9.1%, *p* = 0.29), and the tendency observed at follow-up reflected a significant increase from T0 to T1 among co-twins (hypertension from 6 to 16, *p* = 0.002; cardiovascular disease from 3 to 11, *p* = 0.021; dyslipidaemia from 4 to 12, *p* = 0.021), whereas no significant change occurred in affected twins. Finally, lifetime exposure to solvents/pollutants was significantly more frequent in affected twins (*p* = 0.031) and, in every discordant pair, was confined to the affected member; pesticide exposure followed the same direction without reaching significance. However, these exposures were reported retrospectively at T1, when disease status was already known, so that differential recall cannot be excluded, and the difference in solvent/pollutant exposure did not survive FDR correction (q = 0.062). Whole-cohort and MZ subgroup data are reported together in Table 2.

### 3.3. Nutritional Status

Nutritional status, assessed by body weight and BMI, was similar within pairs. At baseline, neither weight nor BMI differed between affected and unaffected co-twins, irrespective of zygosity; the same was true at follow-up and at baseline in the pairs followed up. Mean body weight decreased slightly over the follow-up period in both groups (in the pairs followed up, from 68.8 to 67.3 kg in affected twins, *p* = 0.066, and from 69.2 to 68.1 kg in unaffected co-twins, *p* = 0.45), and nutritional status remained comparable within pairs, indicating that overt malnutrition was not a prominent feature in this relatively well-preserved, community-dwelling cohort of PD-discordant twins, even after a decade of disease progression. Similar results were observed in the MZ subgroup (Table 3). Because at T1 body weight was self-reported and BMI was calculated using baseline height, follow-up comparisons should be regarded as exploratory, and conclusions on nutritional status rely primarily on the objectively measured baseline data.

### 3.4. Dietary Habits

Most dietary habits were similar between affected twins and their unaffected co-twins, with the exception of two patterns. The first is a stably lower intake of preserved fish (canned fish in oil and smoked salmon) in affected twins: the within-pair difference was significant at baseline (*p* = 0.021) and again at follow-up (*p* = 0.004), without reversing direction over the decade (in the pairs followed up, intake was already lower in affected twins at baseline, 0.45 vs. 0.91, *p* = 0.070, and did not change from T0 to T1 in either group); this whole-cohort difference did not, however, reach significance in the smaller monozygotic subgroup. The second pattern is a reversal of fruit intake: fruit was lower in PD at baseline—significantly so within the genetically identical monozygotic pairs (*p* = 0.001)—but higher in PD at follow-up, both overall (*p* = 0.006) and within MZ pairs (*p* = 0.029). Within these maximally genetically controlled pairs, therefore, fruit—not preserved fish—was the dietary variable that differed significantly at both time points. In the pairs followed up, however, fruit intake did not differ between twins at baseline (1.58 vs. 1.52, *p* = 0.77; MZ 0.87 vs. 1.30, *p* = 0.13). From T0 to T1, fruit intake increased in affected twins (from 1.58 to 2.45, *p* = 0.004), but not in co-twins (from 1.52 to 1.68, *p* = 0.41), and, in MZ pairs, the increase was significantly greater in affected twins than in their co-twins (*p* < 0.001). A lower intake of oil/vegetable fats also emerged in PD at follow-up (*p* = 0.035); analysis of intake from T0 to T1 showed an increase among co-twins (*p* = 0.012), rather than a reduction among affected twins. Added-sugar intake was borderline higher in PD at baseline (*p* = 0.050). Water consumption did not differ between affected twins and unaffected co-twins. After FDR correction within the dietary domain, the differences in preserved-fish and fruit intake at follow-up (q = 0.018 for both) and the lower baseline fruit intake in MZ pairs (q = 0.006) remained significant, whereas the baseline difference in preserved fish (q = 0.13), the lower oil/vegetable-fat intake at follow-up (q = 0.070) and the borderline added-sugar difference (q = 0.15) did not and should be regarded as hypothesis-generating (Supplementary Table S3). These findings are summarized in Table 4, which reports the dietary variables assessed at baseline and follow-up, including within-pair comparisons in the whole cohort and in the monozygotic subgroup, together with the baseline values of the pairs followed up.

### 3.5. The Two Converted (Concordant) Pairs

Two of the 26 MZ pairs (7.7%) were excluded from the longitudinal analysis because they converted from discordance to concordance during the 10-year follow-up, when the initially unaffected co-twin developed PD. In the first male pair (both aged 53 years at baseline and 63 years at follow-up), the initially affected twin already had a 15-year disease history at the final assessment, whereas the co-twin had been diagnosed only 6 months earlier—a 14.5-year difference in age at onset. In the second female pair (both aged 69 years at baseline and 79 years at follow-up), the difference was approximately 12 years (16-year versus 4-year disease duration).

## 4. Discussion

To our knowledge, this is the first 10-year longitudinal follow-up of twins discordant for PD, with paired analyses conducted in both the entire cohort and the MZ subgroup, extending the evidence previously available in the literature [20,21,22,25,26,27]. The most clinically relevant finding is the markedly higher 10-year mortality observed among affected twins. This result is reinforced by the analysis of the MZ subgroup, in which every death occurred in the affected twin, indicating that the excess mortality cannot be explained solely by shared genetic background. The conversion to concordance in 2 of the 26 MZ pairs (7.7%) further illustrates that, even among genetically identical individuals, PD may manifest at different time points, supporting the presence of a prolonged and variable preclinical phase of the disease [9,28]. The sex-stratified analysis (Section 3.1) confirmed a significant within-pair mortality disadvantage in both men and women, with a trend toward higher mortality among men in both groups. This finding is consistent with previous studies showing that male patients with PD have a higher risk of mortality than female patients, supporting the hypothesis that male sex is associated with a less favourable prognosis in PD [29,30]. The absence of within-pair differences in nutritional status (body weight and BMI) and in non-neurological comorbidities indicates that, in this cohort, these variables do not behave as sensitive markers of within-pair divergence over a decade. Taken together with the mortality findings, this pattern shows that the mortality difference persisted despite no detected within-pair differences in the measured nutritional and comorbidity variables. This is consistent with a major contribution of PD itself; however, the absence of statistically significant differences does not demonstrate equivalence in a cohort of this size, and unmeasured factors influencing survival (e.g., disease severity, frailty or access to care) cannot be excluded. The tendency toward more frequent hypertension among unaffected co-twins at follow-up, which reflected an increase over time among co-twins rather than a decrease among affected twins, may be related to the hypotensive effect of dopaminergic therapy and to dysautonomia in PD, rather than reflecting a genuine cardiovascular advantage conferred by the disease. Regarding smoking, our findings are consistent with the well-described inverse association between tobacco use and PD risk [1,7]. Active smoking was more frequent among unaffected co-twins at baseline, a difference that was not confirmed in the follow-up, when the proportions of active and former smokers were comparable between groups, mainly because some co-twins quit smoking during follow-up. This pattern suggests that smoking exposure may have contributed to the initial distribution of disease status—consistent with a potential protective effect—but does not appear to account for the subsequent divergence in clinical progression or mortality, which more plausibly reflects the disease process itself. Beyond mortality, the observation that constipation was already present at baseline and remained evident after 10 years of follow-up, both in the overall cohort and in the monozygotic subgroup, further supports the hypothesis that enteric nervous system involvement is an early event in PD pathogenesis, in line with the Braak hypothesis [18,19]. The emergence of dysphagia at follow-up, virtually confined to the affected twins, is consistent with the expected evolution of bulbar dysfunction with disease progression and, together with constipation, reinforces the impression that gastrointestinal involvement tracks disease stage rather than shared background factors. The dietary findings add a further layer of interpretation. The persistently lower intake of preserved fish among affected twins points to a consistent but not fully genetically robust dietary pattern: the difference was significant in the whole cohort at both time points but did not reach significance in the smaller, maximally genetically controlled MZ subgroup—precisely where confounding by shared genetic background is minimised—and should therefore be interpreted with caution rather than as an established disease-associated dietary trait. The reversal in fruit intake, by contrast—lower in PD at baseline but higher at follow-up, and significant within the MZ subgroup at both time points—may be more consistent with an acquired behavioural change than with a fixed trait. One possible explanation is adherence to dietary advice received during specialist follow-up, such as increased fibre intake to manage constipation; however, neither exposure to dietary counselling nor the reasons for dietary change were measured, so this interpretation remains speculative. Moreover, because the FFQ was administered during a specialist visit at T0 and by telephone at T1, part of the changes between T0 and T1 may reflect the change in assessment mode; such an effect would be expected to apply to both members of a pair, so that within-pair comparisons at each time point and the comparison of changes between twins are less sensitive to it. Because our FFQ recorded consumption frequency rather than quantities or nutrient composition, the lower preserved-fish intake may also reflect factors other than omega-3 content, such as dietary sodium restriction or general food preference; any link to the anti-inflammatory or neuroprotective properties of omega-3 fatty acids [31] remains unproven. The lower oil and vegetable-fat intake in affected twins at follow-up, which reflected an increase among co-twins rather than a reduction in affected twins and did not survive FDR correction, warrants further evaluation within the broader framework of adherence to the Mediterranean diet [13,15,19,24]. Regarding protein-source foods, exploratory analysis of the non-quantitative FFQ items showed a longitudinal decrease in the frequency of red meat and processed meat consumption and an increase in the frequency of egg and legume consumption over the decade in both affected twins and unaffected co-twins, suggesting a general secular shift in dietary habits rather than a PD-specific change in protein intake or its temporal distribution.

### Strengths and Limitations

Our study has several limitations. First, recruiting and maintaining long-term follow-up in twins discordant for PD is inherently challenging because of the rarity and highly selected nature of this population, resulting in a relatively small sample size and a limited number of deaths; accordingly, HRs, particularly in the MZ subgroup, were estimated with wide CIs. Second, the follow-up cohort is a selected subset of the baseline cohort: deaths, refusal and phenoconversion reduced the sample from 54 to 33 pairs (15 MZ). At baseline, retained pairs were younger and, among affected twins, had shorter disease duration and lower levodopa dose than pairs not retained (Supplementary Table S1). Conditioning on survival and persistent discordance may therefore have selected affected twins with milder or slower disease, which may have attenuated within-pair differences at T1 and limits the generalizability of the longitudinal findings; for this reason, changes over time were assessed only within the same 33 pairs. Third, the assessment mode differed between time points: at T0 anthropometry was measured directly and the FFQ was administered during a specialist visit, whereas at T1 data were collected by telephone. Body weight at T1 was self-reported and BMI was calculated using baseline height; because self-reported weight tends to be under-reported and baseline height is likely to overestimate current height given age- and PD-related postural changes, T1 BMI is probably underestimated, possibly more in affected twins. T1 anthropometric and dietary comparisons should therefore be regarded as exploratory, and conclusions on nutritional status rely primarily on the objectively measured baseline data. Fourth, the FFQ was non-quantitative, recording consumption frequency rather than portion size or nutrient intake, and exposure to dietary counselling was not recorded. Fifth, comorbidities were assessed primarily from clinical history rather than through systematic clinical verification, and environmental exposures were collected retrospectively at T1, when disease status was already known, so that misclassification and recall bias cannot be excluded. Finally, numerous secondary comparisons were performed; although FDR correction was applied within domains, none of the secondary comparisons remained significant when correction was applied across all 78 tests, so that these analyses are exploratory and isolated nominal associations may have arisen by chance. Nevertheless, the discordant twin design remains a unique methodological strength: by controlling for shared genetic background and early-life environment over a 10-year follow-up, it provides an exceptional opportunity to disentangle disease-related effects from familial factors.

## 5. Conclusions

In this 10-year longitudinal study of PD-discordant twins, affected twins showed markedly higher mortality than their unaffected co-twins, and in the MZ subgroup all deaths occurred in the affected twin, indicating that the excess mortality cannot be explained by shared genetic background alone. The mortality difference persisted despite no detected within-pair differences in BMI and comorbidities, which is consistent with a major contribution of the disease process itself and of non-shared environmental and stochastic influences accumulated throughout life, although other unmeasured determinants of survival cannot be excluded. Constipation was the most consistent clinical difference and worsened over time in affected twins, whereas dietary differences were limited and partly time-dependent and should be regarded as exploratory. Future studies should involve larger, multicentre prospective twin cohorts with objective anthropometric measurements and quantitative dietary assessment at each time point, standardized ascertainment of the causes of death, disease severity and frailty, and the integration of biological markers (e.g., gut microbiota, epigenetic and molecular profiles), in order to clarify the mechanisms linking PD progression to survival and to identify modifiable targets for intervention.

## Figures and Tables

**Table 1:** Mortality and 10-year survival in the whole cohort (54 pairs).

Mortality/Survival	PD	Unaffected Co-Twins	*p*
Deaths, total	17 (31.5%)	3 (5.6%)	**0.0005**
in MZ pairs	9	0	—
in DZ pairs	8	3	—
5-year survival (KM)	79.2%	98.2%	—
10-year survival (KM)	65.5%	94.5%	**<0.001**
10-year survival, MZ pairs (KM)	61.4%	100.0%	**<0.001**
Cox HR (PD vs. co-twin)	6.83 (2.00–23.31)	—	**0.002**
Cox HR (PD vs. co-twin), MZ subgroup ^1^	22.95 (2.90–2962.10)	—	**0.0007**

McNemar test for the within-pair comparison of deaths; Kaplan–Meier method and log-rank test for survival; Cox regression for the hazard ratio. ^1^ In the MZ subgroup all deaths occurred in the affected twin (0 events among co-twins), so the standard Cox partial likelihood is not estimable (complete separation); the HR and *p*-value shown are from a Firth (penalized-likelihood) Cox model, which yields a finite estimate; the very wide CI indicates that the magnitude of the effect is highly uncertain. A model-free sign test on the 9 MZ pairs discordant for death (in all of which the affected twin died) supports the same direction (exact two-sided *p* = 0.0039). Sensitivity analyses accounting for the matched design are reported in Section 3.1. Significant *p*-values are shown in bold. PD, Parkinson’s disease (affected twins); MZ, monozygotic; DZ, dizygotic; KM, Kaplan–Meier; HR, hazard ratio; CI, confidence interval.

**Table 2:** Clinical, lifestyle, metabolic and environmental features in the whole cohort (overall, 54 pairs) and in the monozygotic (MZ) subgroup at baseline (T0) and follow-up (T1). Baseline values of the 33 pairs reassessed at follow-up (15 MZ) are also reported.

Variable		Whole Cohort—Overall (54 Pairs/26 MZ)			Baseline T0 of the Pairs Followed Up (33 Pairs/15 MZ)			Follow-Up T1 (33 Pairs/15 MZ)		
		PD	Co-Twin	*p*	PD	Co-Twin	*p*	PD	Co-Twin	*p*
Disease duration, years	Overall	7.1 ± 5.4	0	—	4.9 ± 4.2	0	—	15.2 ± 5.2	0	—
	MZ	8.2 ± 6.0	0	—	6.4 ± 5.4	0	—	16.2 ± 6.2	0	—
Levodopa, mg/day	Overall	340.3 ± 272.1	0	—	293.9 ± 239.7	0	—	433.3 ± 257.7	0	—
	MZ	398.1 ± 255	0	—	330.0 ± 284.0	0	—	480.8 ± 250	0	—
Smoking										
Active smokers	Overall	4 (7.4%)	8 (14.8%)	0.22	2 (6.1%)	4 (12.1%)	0.50	2 (6.1%)	2 (6.1%)	1.00
	MZ	3 (11.5%)	4 (15.4%)	1.00	1 (6.7%)	2 (13.3%)	1.00	1 (6.7%)	2 (13.3%)	1.00
Former smokers	Overall	14 (25.9%)	12 (22.2%)	0.75	7 (21.2%)	8 (24.2%)	1.00	9 (27.3%)	11 (33.3%)	0.69
	MZ	5 (19.2%)	5 (19.2%)	1.00	2 (13.3%)	2 (13.3%)	1.00	4 (26.7%)	2 (13.3%)	0.50
Never smokers	Overall	36 (66.7%)	34 (63.0%)	0.79	24 (72.7%)	21 (63.6%)	0.38	22 (66.7%)	20 (60.6%)	0.69
	MZ	18 (69.2%)	17 (65.4%)	1.00	12 (80.0%)	11 (73.3%)	1.00	10 (66.7%)	11 (73.3%)	1.00
Metabolic comorbidities										
Diabetes	Overall	3 (5.6%)	2 (3.7%)	1.00	1 (3.0%)	0 (0.0%)	1.00	2 (6.1%)	1 (3.0%)	1.00
	MZ	1 (3.8%)	2 (7.7%)	1.00	0 (0.0%)	0 (0.0%)	—	1 (6.7%)	1 (6.7%)	1.00
Dyslipidemia	Overall	6 (11.1%)	9 (16.7%)	0.75	3 (9.1%)	4 (12.1%)	1.00	8 (24.2%)	12 (36.4%)	0.39
	MZ	2 (7.7%)	5 (19.2%)	0.45	0 (0.0%)	1 (6.7%)	1.00	4 (26.7%)	6 (40.0%)	0.69
Cardiovascular	Overall	14 (25.9%)	9 (16.7%)	0.27	7 (21.2%)	3 (9.1%)	0.29	9 (27.3%)	11 (33.3%)	0.73
	MZ	6 (23.1%)	3 (11.5%)	0.38	1 (6.7%)	0 (0.0%)	1.00	2 (13.3%)	5 (33.3%)	0.25
Hypertension	Overall	17 (31.5%)	15 (27.8%)	0.77	8 (24.2%)	6 (18.2%)	0.63	10 (30.3%)	16 (48.5%)	0.109
	MZ	6 (23.1%)	5 (19.2%)	1.00	1 (6.7%)	0 (0.0%)	1.00	2 (13.3%)	6 (40.0%)	0.22
Thyroid disorders	Overall	4 (7.4%)	3 (5.6%)	1.00	2 (6.1%)	2 (6.1%)	1.00	4 (12.1%)	4 (12.1%)	1.00
	MZ	2 (7.7%)	1 (3.8%)	1.00	0 (0.0%)	0 (0.0%)	—	2 (13.3%)	1 (6.7%)	1.00
Gastrointestinal										
Constipation	Overall	20 (37.0%)	6 (11.1%)	**0.004**	9 (27.3%)	4 (12.1%)	0.27	16 (48.5%)	8 (24.2%)	**0.011**
	MZ	9 (34.6%)	3 (11.5%)	0.15	5 (33.3%)	2 (13.3%)	0.45	9 (60.0%)	3 (20.0%)	**0.031**
Regular bowel habit	Overall	32 (59.3%)	46 (85.2%)	**0.008**	22 (66.7%)	28 (84.8%)	0.15	14 (42.4%)	22 (66.7%)	**0.021**
	MZ	17 (65.4%)	23 (88.5%)	0.15	10 (66.7%)	13 (86.7%)	0.45	4 (26.7%)	11 (73.3%)	**0.016**
Dysphagia	Overall	0	0	—	0	0	—	5 (15.2%)	1 (3.0%)	0.125
	MZ	0	0	—	0	0	—	1 (6.7%)	0 (0%)	1.00
Environmental exposure										
Solvents/pollutants	Overall	—	—	—	—	—	—	9 (27.3%)	3 (9.1%)	**0.031**
	MZ	—	—	—	—	—	—	4 (26.7%)	1 (6.7%)	0.25
Pesticides	Overall	—	—	—	—	—	—	3 (9.1%)	0 (0%)	0.25
	MZ	—	—	—	—	—	—	2 (13.3%)	0 (0%)	0.50

n (%) or mean ± SD. McNemar test for paired categorical data (exact binomial McNemar used for the MZ subgroup and other sparse cells); environmental exposure (solvents/pollutants, pesticides) assessed at T1 only. Notably, within MZ pairs a regular bowel habit was significantly less common in the affected twin at follow-up (26.7% vs. 73.3%; *p* = 0.016), mirroring the constipation finding above. Significant *p*-values are shown in bold. Middle columns: baseline values of the pairs reassessed at T1 (exact McNemar test for categorical variables). Changes from T0 to T1 within each group are reported in the text. Nominal *p*-values are shown; Benjamini–Hochberg FDR-adjusted q-values are reported in Supplementary Table S3. PD, affected twin; Co-twin, unaffected co-twin; MZ, monozygotic; T0, baseline; T1, 10-year follow-up; FDR, false discovery rate.

**Table 3:** Nutritional status in the whole cohort and in the MZ subgroup at baseline (T0) and follow-up (T1). Baseline values of the 33 pairs reassessed at follow-up (15 MZ) are also reported.

Variable		Whole Cohort—Overall (54 Pairs/26 MZ)			Baseline T0 of the Pairs Followed Up (33 Pairs/15 MZ)			Follow-Up T1 (33 Pairs/15 MZ)		
		PD	Co-Twin	*p*	PD	Co-Twin	*p*	PD	Co-Twin	*p*
Weight, kg	Overall	68.2 ± 15.9	69.7 ± 15.8	0.36	68.8 ± 12.5	69.2 ± 12.9	0.84	67.3 ± 13.6	68.1 ± 13.3	0.33
	MZ	69.0 ± 19.0	73.0 ± 18.5	0.069	69.2 ± 11.8	72.3 ± 12.6	0.13	68.3 ± 10.8	71.3 ± 12.9	0.20
BMI, kg/m^2^	Overall	24.5 ± 5.2	25.0 ± 4.7	0.37	24.4 ± 4.0	24.8 ± 3.9	0.50	24.5 ± 3.9	24.6 ± 3.7	0.46
	MZ	24.4 ± 5.9	25.4 ± 5.1	0.22	24.1 ± 2.5	24.8 ± 3.1	0.32	24.1 ± 2.1	24.7 ± 3.3	0.46

Mean ± SD. Body weight and BMI did not differ within pairs at either time point, including in the MZ subgroup. Middle columns: baseline values of the pairs reassessed at T1 (paired *t*-test). Changes from T0 to T1 within each group are reported in the text. At T1, body weight was self-reported and BMI was calculated using baseline height; T1 values should be regarded as exploratory. BMI, body mass index; PD, affected twin; Co-twin, unaffected co-twin; MZ, monozygotic.

**Table 4:** Dietary habits (FFQ, consumption-frequency units) in the whole cohort and in the MZ subgroup at baseline (T0) and follow-up (T1). Baseline values of the 33 pairs reassessed at follow-up (15 MZ) are also reported.

Food Item		Whole Cohort—Overall (54 Pairs/26 MZ)			Baseline T0 of the Pairs Followed Up (33 Pairs/15 MZ)			Follow-Up T1 (33 Pairs/15 MZ)		
		PD	Co-Twin	*p*	PD	Co-Twin	*p*	PD	Co-Twin	*p*
Preserved fish	Overall	0.54 ± 0.74	1.04 ± 1.29	**0.021**	0.45 ± 0.71	0.91 ± 0.98	0.070	0.48 ± 0.62	1.03 ± 0.81	**0.004**
	MZ	0.52 ± 0.77	1.12 ± 1.67	0.134	0.53 ± 0.83	0.87 ± 1.06	0.43	0.67 ± 0.72	1.07 ± 0.88	0.164
Fruit	Overall	1.49 ± 1.03	1.75 ± 1.12	0.17	1.58 ± 1.09	1.52 ± 1.18	0.77	2.45 ± 1.43	1.68 ± 1.17	**0.006**
	MZ	0.90 ± 0.76	1.72 ± 1.17	**0.001**	0.87 ± 0.74	1.30 ± 1.32	0.13	2.27 ± 1.57	1.37 ± 1.26	**0.029**
Vegetables	Overall	1.54 ± 0.60	1.69 ± 0.74	0.15	1.45 ± 0.62	1.59 ± 0.75	0.29	2.10 ± 1.10	1.95 ± 0.93	0.55
	MZ	1.34 ± 0.55	1.56 ± 0.75	0.20	1.23 ± 0.53	1.43 ± 0.82	0.38	1.67 ± 1.03	1.60 ± 1.00	0.86
Oil/vegetable fats	Overall	4.16 ± 1.75	4.18 ± 2.08	0.95	3.82 ± 1.82	3.85 ± 1.91	0.93	4.12 ± 1.43	4.91 ± 1.51	**0.035**
	MZ	3.80 ± 1.65	4.04 ± 1.66	0.55	3.47 ± 1.72	3.67 ± 2.08	0.74	4.13 ± 1.64	4.87 ± 1.46	0.23
Added sugars	Overall	0.93 ± 1.43	0.53 ± 0.81	0.050	0.85 ± 1.44	0.50 ± 0.74	0.17	0.94 ± 1.04	0.61 ± 1.02	0.17
	MZ	1.06 ± 1.68	0.58 ± 0.70	0.14	0.83 ± 1.53	0.70 ± 0.80	0.71	0.97 ± 1.01	0.73 ± 1.16	0.55
Water	Overall	1.01 ± 0.48	0.99 ± 0.53	0.81	0.94 ± 0.50	1.00 ± 0.52	0.52	1.21 ± 0.64	1.17 ± 0.51	0.72
	MZ	1.02 ± 0.46	0.98 ± 0.57	0.74	0.97 ± 0.44	0.87 ± 0.52	0.46	1.20 ± 0.59	1.07 ± 0.50	0.33

Mean ± SD; within-pair paired t-test. Preserved-fish intake was stably lower in PD, whereas fruit intake changed from lower (T0) to higher (T1). In the MZ (genetically identical) discordant pairs (26 at T0, 15 at T1), the maximally genetically controlled subgroup, the significant dietary signal was fruit—lower in PD at baseline (0.90 vs. 1.72; *p* = 0.001) and higher at follow-up (2.27 vs. 1.37; *p* = 0.029)—mirroring the whole-cohort reversal; the lower preserved-fish intake seen in the whole cohort did not reach significance in the smaller MZ subgroup. Significant *p*-values are shown in bold. Middle columns: baseline values of the pairs reassessed at T1 (paired t-test). Changes from T0 to T1 within each group are reported in the text. Preserved fish: canned fish in oil (tuna, mackerel, sardines) and smoked salmon. At T1, the FFQ was administered by telephone interview, and T1 dietary comparisons should be regarded as exploratory. Nominal *p*-values are shown; FDR-adjusted q-values are reported in Supplementary Table S3. FFQ, food frequency questionnaire; PD, affected twin; Co-twin, unaffected co-twin; MZ, monozygotic; FDR, false discovery rate.

## Data Availability

The datasets generated and/or analysed during the current study are available from the corresponding author upon reasonable request. Given the rarity of the discordant-twin cohort and the consequent re-identification risk, individual-level data are not deposited in a public repository.

## Supplementary Materials

The following supporting information can be downloaded at: https://www.mdpi.com/article/10.3390/brainsci16101102/s1, Table S1: Baseline characteristics of the 33 pairs retained at follow-up versus the 21 pairs not retained. Table S2: Sensitivity analysis—within-pair *p*-values for every continuous/FFQ variable computed with the paired Student’s *t*-test and the Wilcoxon signed-rank test (whole cohort and MZ subgroup), at baseline (T0) and follow-up (T1). All within-pair comparisons of continuous and FFQ variables in this manuscript use the paired Student’s *t*-test, applied uniformly at T0 and T1; every such comparison was repeated with the non-parametric Wilcoxon signed-rank test as a sensitivity analysis, with concordant significance status for all reported variables except two borderline baseline comparisons (added sugars in the whole cohort: *t*-test *p* = 0.050, Wilcoxon *p* = 0.047; body weight in the MZ subgroup: *t*-test *p* = 0.069, Wilcoxon *p* = 0.032; see Table S2 for details). Table S3: Within-pair comparisons of secondary endpoints with nominal *p*-values and Benjamini–Hochberg FDR-adjusted q-values.

## Abbreviations

The following abbreviations are used in this manuscript:

AED	electronic digital archive
BMI	body mass index
CI	confidence interval
DZ	dizygotic
FDR	false discovery rate
FFQ	food frequency questionnaire
HR	hazard ratio
KM	Kaplan–Meier
MZ	monozygotic
PD	Parkinson’s disease
SD	standard deviation
T0	baseline assessment
T1	10-year follow-up assessment
