# Supplementary material for: Twins Discordant for Parkinson’s Disease: A 10-Year Longitudinal Follow-Up of Mortality, Clinical and Lifestyle Features, Nutritional Status, and Dietary Habits
Source: Brain Sci. 2026 Oct 13;16(10):1102. doi: 10.3390/brainsci16101102 (PMC13702638; doi:10.3390/brainsci16101102)
Supplement: Supplementary file 1 [file brainsci-16-01102-s001.zip › brainsci-4583133-supplementary.pdf]

**Supplementary Materials**

**Supplementary Table S1. Baseline (T0) characteristics of the 33 pairs retained at follow-up versus the 21 pairs not retained.**

| Variable | Retained (33 pairs) | Not retained (21 pairs) | p |
|---|---|---|---|
| **Pairs, n** | 33 | 21 | — |
| **MZ pairs** | 15 (45.5%) | 11 (52.4%) | 0.78 |
| **Age at baseline, years (all twins)** | 60.55 ± 9.42 | 72.67 ± 7.99 | <0.001 |
| ***Affected twins*** | | | |
| **Men** | 19 (57.6%) | 13 (61.9%) | 0.78 |
| **Disease duration, years** | 4.9 ± 4.2 | 9.0 ± 6.3 | 0.013 |
| **Levodopa, mg/day** | 293.9 ± 239.7 | 516.7 ± 244.2 | 0.002 |
| **BMI, kg/m²** | 24.4 ± 4.0 | 24.7 ± 6.8 | 0.88 |
| **Constipation** | 9 (27.3%) | 11 (52.4%) | 0.085 |
| **Active smokers** | 2 (6.1%) | 2 (9.5%) | 0.64 |
| **Cardiovascular disease** | 7 (21.2%) | 7 (33.3%) | 0.36 |
| **Hypertension** | 8 (24.2%) | 9 (42.9%) | 0.23 |
| **Preserved fish (FFQ)** | 0.45 ± 0.71 | 0.67 ± 0.80 | 0.33 |
| **Fruit (FFQ)** | 1.58 ± 1.09 | 1.36 ± 0.98 | 0.45 |
| ***Unaffected co-twins*** | | | |
| **BMI, kg/m²** | 24.8 ± 3.9 | 25.3 ± 5.8 | 0.76 |
| **Constipation** | 4 (12.1%) | 2 (9.5%) | 1.00 |
| **Active smokers** | 4 (12.1%) | 4 (19.0%) | 0.70 |
| **Cardiovascular disease** | 3 (9.1%) | 6 (28.6%) | 0.13 |
| **Hypertension** | 6 (18.2%) | 9 (42.9%) | 0.065 |
| **Preserved fish (FFQ)** | 0.91 ± 0.98 | 1.24 ± 1.70 | 0.43 |
| **Fruit (FFQ)** | 1.52 ± 1.18 | 2.12 ± 0.97 | 0.046 |

Mean ± SD or n (%). Not retained: 18 pairs in which at least one twin died, 1 pair that declined participation and 2 MZ pairs excluded because of phenoconversion of the co-twin. Twins of the same pair share age at baseline. Welch's t-test for continuous variables and Fisher's exact test for categorical variables. Levodopa: affected twins not treated with levodopa counted as 0 mg/day. Preserved fish and fruit in FFQ consumption-frequency units. BMI, body mass index; FFQ, food frequency questionnaire; MZ, monozygotic; SD, standard deviation.

All within-pair comparisons of continuous and FFQ variables in this manuscript use the paired Student's t-test, applied uniformly at T0 and T1. To demonstrate that this methodological choice does not drive any conclusion, every such comparison was repeated with the non-parametric Wilcoxon signed-rank test. As shown in Supplementary Table S2, the significance status is identical under the two tests for all comparisons except two cells, both at the boundary and neither

affecting a reported result: added sugars at T0 (t-test p=0.050 vs Wilcoxon p=0.047, i.e. borderline either way) and weight at T0 in the MZ subgroup (t-test 0.069 vs Wilcoxon 0.032 — a comparison that is not a study finding, since nutritional status did not differ within pairs). This confirms the robustness of the analysis to the parametric/non-parametric choice and supports the use of a single uniform test throughout.

**Supplementary Table S2. Sensitivity analysis — within-pair p-values for every continuous/FFQ variable computed with the paired Student's t-test and the Wilcoxon signed-rank test (whole cohort and MZ subgroup), at baseline (T0) and follow-up (T1).**

| Variable | | Baseline T0 (54 pairs / 26 MZ) | | Follow-up T1 (33 pairs / 15 MZ) | |
|---|---|---|---|---|---|
| | | t-test | Wilcoxon | t-test | Wilcoxon |
| **Weight** | Overall | 0.360 | 0.299 | 0.334 | 0.343 |
| | MZ | 0.069 | **0.032** | 0.198 | 0.267 |
| **BMI** | Overall | 0.374 | 0.239 | 0.465 | 0.257 |
| | MZ | 0.215 | 0.134 | 0.465 | 0.720 |
| **Preserved fish** | Overall | **0.021** | **0.026** | **0.004** | **0.005** |
| | MZ | 0.134 | 0.127 | 0.164 | 0.153 |
| **Fresh fish** | Overall | 0.485 | 0.342 | 0.712 | 0.693 |
| | MZ | 0.937 | 0.983 | 0.876 | 0.841 |
| **Fruit** | Overall | 0.165 | 0.137 | **0.006** | **0.009** |
| | MZ | **0.001** | **0.002** | **0.029** | **0.028** |
| **Vegetables** | Overall | 0.145 | 0.211 | 0.552 | 0.550 |
| | MZ | 0.197 | 0.150 | 0.855 | 0.864 |
| **Oil/vegetable fats** | Overall | 0.951 | 0.616 | **0.035** | **0.040** |
| | MZ | 0.553 | 0.574 | 0.228 | 0.220 |
| **Added sugars** | Overall | 0.050 | **0.047** | 0.171 | 0.121 |
| | MZ | 0.141 | 0.158 | 0.550 | 0.427 |

*Two-sided p-values. Every comparison keeps the same significance status under both tests, except the two highlighted cells (added sugars T0, borderline; weight T0 in MZ, not a study finding). Categorical variables were analysed with McNemar and survival with log-rank/Cox, independently of this choice.*

**Supplementary Table S3. Within-pair comparisons of secondary endpoints: nominal p-values and Benjamini–Hochberg false discovery rate (FDR)-adjusted q-values.**

| Variable | Time | Pairs | Nominal p | q (within domain) | q (all 78 tests) |
|---|---|---|---|---|---|
| ***Smoking*** | | | | | |
| **Active smokers** | T0 | Overall | 0.22 | 0.66 | 0.57 |
| **Active smokers** | T0 | MZ | 1.00 | 1.00 | 1.00 |
| **Former smokers** | T0 | Overall | 0.75 | 0.79 | 1.00 |

| Variable | Time | Pairs | Nominal p | q (within domain) | q (all 78 tests) |
|---|---|---|---|---|---|
| **Former smokers** | T0 | MZ | 1.00 | 1.00 | 1.00 |
| **Never smokers** | T0 | Overall | 0.79 | 0.79 | 1.00 |
| **Never smokers** | T0 | MZ | 1.00 | 1.00 | 1.00 |
| **Active smokers** | T1 | Overall | 1.00 | 1.00 | 1.00 |
| **Active smokers** | T1 | MZ | 1.00 | 1.00 | 1.00 |
| **Former smokers** | T1 | Overall | 0.69 | 1.00 | 1.00 |
| **Former smokers** | T1 | MZ | 0.50 | 1.00 | 0.85 |
| **Never smokers** | T1 | Overall | 0.69 | 1.00 | 1.00 |
| **Never smokers** | T1 | MZ | 1.00 | 1.00 | 1.00 |
| ***Metabolic comorbidities*** | | | | | |
| **Diabetes** | T0 | Overall | 1.00 | 1.00 | 1.00 |
| **Diabetes** | T0 | MZ | 1.00 | 1.00 | 1.00 |
| **Dyslipidemia** | T0 | Overall | 0.75 | 1.00 | 1.00 |
| **Dyslipidemia** | T0 | MZ | 0.45 | 1.00 | 0.82 |
| **Cardiovascular** | T0 | Overall | 0.27 | 1.00 | 0.60 |
| **Cardiovascular** | T0 | MZ | 0.38 | 1.00 | 0.74 |
| **Hypertension** | T0 | Overall | 0.77 | 1.00 | 1.00 |
| **Hypertension** | T0 | MZ | 1.00 | 1.00 | 1.00 |
| **Thyroid disorders** | T0 | Overall | 1.00 | 1.00 | 1.00 |
| **Thyroid disorders** | T0 | MZ | 1.00 | 1.00 | 1.00 |
| **Diabetes** | T1 | Overall | 1.00 | 1.00 | 1.00 |
| **Diabetes** | T1 | MZ | 1.00 | 1.00 | 1.00 |
| **Dyslipidemia** | T1 | Overall | 0.39 | 0.98 | 0.74 |
| **Dyslipidemia** | T1 | MZ | 0.69 | 1.00 | 1.00 |
| **Cardiovascular** | T1 | Overall | 0.73 | 1.00 | 1.00 |
| **Cardiovascular** | T1 | MZ | 0.25 | 0.63 | 0.57 |
| **Hypertension** | T1 | Overall | 0.109 | 0.55 | 0.53 |
| **Hypertension** | T1 | MZ | 0.22 | 0.63 | 0.57 |
| **Thyroid disorders** | T1 | Overall | 1.00 | 1.00 | 1.00 |
| **Thyroid disorders** | T1 | MZ | 1.00 | 1.00 | 1.00 |
| ***Gastrointestinal*** | | | | | |

| Variable | Time | Pairs | Nominal p | q (within domain) | q (all 78 tests) |
|---|---|---|---|---|---|
| **Constipation** | T0 | Overall | 0.004 | 0.008 | 0.10 |
| **Constipation** | T0 | MZ | 0.15 | 0.15 | 0.53 |
| **Regular bowel habit** | T0 | Overall | 0.008 | 0.008 | 0.12 |
| **Regular bowel habit** | T0 | MZ | 0.15 | 0.15 | 0.53 |
| **Constipation** | T1 | Overall | 0.011 | 0.032 | 0.14 |
| **Constipation** | T1 | MZ | 0.031 | 0.047 | 0.20 |
| **Regular bowel habit** | T1 | Overall | 0.021 | 0.032 | 0.18 |
| **Regular bowel habit** | T1 | MZ | 0.016 | 0.047 | 0.18 |
| **Dysphagia** | T1 | Overall | 0.125 | 0.13 | 0.53 |
| **Dysphagia** | T1 | MZ | 1.00 | 1.00 | 1.00 |
| ***Environmental exposure*** | | | | | |
| **Solvents/pollutants** | T1 | Overall | 0.031 | 0.062 | 0.20 |
| **Solvents/pollutants** | T1 | MZ | 0.25 | 0.50 | 0.57 |
| **Pesticides** | T1 | Overall | 0.25 | 0.25 | 0.57 |
| **Pesticides** | T1 | MZ | 0.50 | 0.50 | 0.85 |
| ***Nutritional status*** | | | | | |
| **Weight, kg** | T0 | Overall | 0.36 | 0.37 | 0.74 |
| **Weight, kg** | T0 | MZ | 0.069 | 0.14 | 0.36 |
| **BMI, kg/m²** | T0 | Overall | 0.37 | 0.37 | 0.74 |
| **BMI, kg/m²** | T0 | MZ | 0.22 | 0.22 | 0.57 |
| **Weight, kg** | T1 | Overall | 0.33 | 0.46 | 0.70 |
| **Weight, kg** | T1 | MZ | 0.20 | 0.40 | 0.57 |
| **BMI, kg/m²** | T1 | Overall | 0.46 | 0.46 | 0.82 |
| **BMI, kg/m²** | T1 | MZ | 0.46 | 0.46 | 0.82 |
| ***Dietary habits*** | | | | | |
| **Preserved fish** | T0 | Overall | 0.021 | 0.13 | 0.18 |
| **Preserved fish** | T0 | MZ | 0.134 | 0.28 | 0.53 |
| **Fruit** | T0 | Overall | 0.17 | 0.26 | 0.53 |
| **Fruit** | T0 | MZ | 0.001 | 0.006 | 0.078 |
| **Vegetables** | T0 | Overall | 0.15 | 0.26 | 0.53 |
| **Vegetables** | T0 | MZ | 0.20 | 0.30 | 0.57 |

| Variable | Time | Pairs | Nominal p | q (within domain) | q (all 78 tests) |
|---|---|---|---|---|---|
| **Oil/vegetable fats** | T0 | Overall | 0.95 | 0.95 | 1.00 |
| **Oil/vegetable fats** | T0 | MZ | 0.55 | 0.66 | 0.88 |
| **Added sugars** | T0 | Overall | 0.050 | 0.15 | 0.28 |
| **Added sugars** | T0 | MZ | 0.14 | 0.28 | 0.53 |
| **Water** | T0 | Overall | 0.81 | 0.95 | 1.00 |
| **Water** | T0 | MZ | 0.74 | 0.74 | 1.00 |
| **Preserved fish** | T1 | Overall | 0.004 | 0.018 | 0.10 |
| **Preserved fish** | T1 | MZ | 0.164 | 0.46 | 0.53 |
| **Fruit** | T1 | Overall | 0.006 | 0.018 | 0.12 |
| **Fruit** | T1 | MZ | 0.029 | 0.17 | 0.20 |
| **Vegetables** | T1 | Overall | 0.55 | 0.66 | 0.88 |
| **Vegetables** | T1 | MZ | 0.86 | 0.86 | 1.00 |
| **Oil/vegetable fats** | T1 | Overall | 0.035 | 0.070 | 0.21 |
| **Oil/vegetable fats** | T1 | MZ | 0.23 | 0.46 | 0.57 |
| **Added sugars** | T1 | Overall | 0.17 | 0.26 | 0.53 |
| **Added sugars** | T1 | MZ | 0.55 | 0.66 | 0.88 |
| **Water** | T1 | Overall | 0.72 | 0.72 | 1.00 |
| **Water** | T1 | MZ | 0.33 | 0.50 | 0.70 |

Nominal p-values are those reported in Tables 2–4 (paired Student's t-test for continuous and FFQ variables; McNemar test for categorical variables). q (within domain): Benjamini–Hochberg FDR correction applied within each domain, separately for each time point (T0, 54 pairs; T1, 33 pairs) and subgroup (Overall; MZ). q (all 78 tests): correction applied across all within-pair comparisons of secondary endpoints reported in Tables 2–4. The baseline values of the 33 pairs reassessed at follow-up (middle columns of Tables 2–4) and within-group changes are descriptive and were not included. Preserved fish: canned fish in oil (tuna, mackerel, sardines) and smoked salmon. FDR, false discovery rate; FFQ, food frequency questionnaire; MZ, monozygotic; T0, baseline; T1, 10-year follow-up.
